# Predicting Early Unplanned Readmission to Critical Care Units Using Machine Learning and Metaheuristic Hyperparameter Optimization: Evidence from a Chilean Public Hospital

**DOI:** 10.3390/jcm15218328

**Published:** 2026-10-26

**Authors:** Fredy Barriga-Gallegos, Gonzalo Ríos-Vásquez, Antonia Ilabaca, Hanns de la Fuente-Mella, Verónica Badilla-Morales, Naldy Febré Vergara

**Affiliations:** 1 Institute for Health Care Research, Faculty of Nursing, Universidad Andrés Bello, Santiago 8370146, Chile; fredy.barriga@unab.cl (F.B.-G.); veribadmor@gmail.com (V.B.-M.); 2 Transportation and Logistics Center, Department of Engineering Sciences, Universidad Andrés Bello, Santiago 7500735, Chile; 3 Departamento de Ingeniería Industrial, Universidad del Bío-Bío, Concepción 4051381, Chile; grios@ubiobio.cl; 4 Instituto de Estadística, Pontificia Universidad Católica de Valparaíso, Valparaíso 2340031, Chile; hanns.delafuente@pucv.cl

**Keywords:** readmission risk, predictive analytics, metaheuristic optimization, health analytics, machine learning

## Abstract

**Background**: Early unplanned readmission to critical care units is associated with adverse outcomes and consumes scarce capacity twice, yet the decision to transfer a patient out of the unit is still made without objective criteria. Machine learning may support this decision, but metaheuristic hyperparameter search remains largely unexplored here. **Methods**: We analyzed 371 patients discharged from the critical care unit of a Chilean public hospital, of whom 41 were readmitted within seven days of transfer to a general ward. Five models (logistic regression, support vector classification, decision tree, random forest, and XGBoost) were trained on clinical and nursing variables available at transfer, using nested stratified five-fold cross-validation with preprocessing fitted within the training folds. Bayesian optimization and a genetic algorithm were compared under identical folds. **Results**: Under nested cross-validation, discrimination was moderate (AUC 53.3–75.2%), and no single model performed best across all metrics. Logistic regression achieved the highest AUC (75.2%) with the lowest variability across folds, followed by XGBoost (75.0%), whose results varied markedly across folds. The genetic algorithm yielded higher AUC than Bayesian optimization for most tree-based models. Days in the critical care unit, age, and unit of origin ranked highest in impurity-based importance. **Conclusions**: This proof-of-concept study suggests that routinely collected clinical and nursing information available at transfer may inform early readmission prediction. As the sample was case-enriched and derived from a single center, recalibration to the natural readmission prevalence and prospective external validation are required before clinical implementation.

## 1. Introduction

Health systems must deliver clinical effectiveness while operating under finite capacity, and this tension is most acute in critical care. In the Chilean public system, critical care is organized as a single functional structure, the *Unidad de Paciente Crítico* (UPC), comprising intensive care beds for patients requiring advanced organ support and intermediate care beds for patients requiring continuous monitoring [1]. The UPC accounts for a small share of hospital beds but a disproportionate share of hospital costs: reported daily costs per patient across OECD countries range from EUR 201 to EUR 4,322, with human resources consistently the largest component [2,3]. This dependence on specialized personnel also makes critical care capacity largely inelastic, since a bed is operational only when adequately staffed. With critical care units internationally reporting routine occupancy near 90% [4], competing admission demands are managed through a single mechanism: the discharge decision [5].

Early readmission is defined as an unplanned return to the same critical care unit after transfer to a lower-acuity ward during the same hospitalization, commonly operationalized within 48 hours to seven days of transfer [6,7]. This paper adopts a seven-day window. The transition is itself a period of vulnerability: adverse events are common after the move from critical care to the general ward [8], and the patient’s residual nursing complexity at transfer independently predicts them [9]. Early readmission is a multifactorial phenomenon. It reflects patient-related factors such as prior hospital trajectory, comorbidity, and residual organ support, as well as organizational factors such as bed pressure and transfer timing, and care-related factors such as nursing workload and continuity of care after discharge [6,10].

Reported rates of early readmission range from 1.3% to 13.4%, and much of this variation reflects differences in discharge practices rather than case mix [6,11]. Readmitted patients consistently experience higher in-hospital mortality, longer stays in both critical care and the hospital, and greater resource use than patients discharged only once [12,13,14]. The burden operates on two levels. Clinically, readmission marks a patient whose deterioration was not anticipated. Operationally, it consumes the same scarce capacity twice, since the readmitted patient occupies a bed that would otherwise be available for a new admission.

Readmission is widely monitored as an indicator of care quality, although its validity in that role is contested, since variation between hospitals partly reflects case mix and discharge practice rather than quality of care itself [7,15]. Its operational relevance, however, is undisputed. When critical care capacity is strained, patients are discharged sooner, and readmission becomes more likely [16], whereas delaying transfer reduces it [17]. The discharge decision therefore involves two errors of unequal nature: discharging too early exposes the patient to deterioration and an avoidable return to critical care, while retaining an already recovered patient occupies a bed that another critically ill patient needs. Anticipating which patients will deteriorate after transfer is at once a clinical and an operational problem.

Two strategies have been proposed to anticipate deterioration after transfer. The first applies severity and workload scores at discharge, including SOFA, NEWS, MEWS, SWIFT, and NAS, on the rationale that residual physiological instability is the most consistent predictor of readmission [18]. These instruments do yield a risk estimate at the moment of transfer, but their reported discrimination is moderate and, at the low prevalence of readmission, their positive predictive values are necessarily low, which makes them more useful for ruling out deterioration than for identifying the patients who will return [19,20]. The same pattern appears at triage, where a machine learning model separated patients by risk of critical care better than the Emergency Severity Index [21]. As generic instruments, they also reduce the patient’s condition to a single dimension, either residual physiological instability or nursing workload, leaving aside the hospital trajectory and the organizational context that also shape readmission risk [6]. The second strategy relies on critical care transition programs that extend follow-up beyond the unit [22], and therefore acts only once the discharge decision has been made. As a result, discharge decisions in routine practice are still reported to lack objective criteria [4].

Because early readmission arises from multiple interacting and nonlinear factors, machine learning (ML) is well suited to the problem: rather than assuming predefined relationships, it learns them directly from historical records [23]. Model performance remains sensitive to hyperparameter configuration [24], and this sensitivity is accentuated in clinical readmission registries, where class imbalance and moderate sample sizes make performance particularly dependent on calibrating model complexity. In these conditions, exhaustive strategies such as grid search scale poorly with search-space dimensionality [25], whereas metaheuristics handle mixed discrete-continuous spaces naturally and have been proposed as an alternative to conventional tuning [26,27,28].

In this context, this paper develops and compares ML models to predict early readmission to the UPC of a public high-complexity hospital in Viña del Mar, Chile, using routinely collected clinical and nursing records. The contribution of this work is threefold. First, every predictor is available when the transfer decision is made, so the model is designed to support that decision rather than to explain readmission retrospectively. Second, five ML models (decision tree, random forest, extreme gradient boosting, logistic regression, and support vector machines) are compared under two hyperparameter optimization strategies, Bayesian optimization and a genetic algorithm, sharing the same objective function and cross-validation partition; while Bayesian optimization has been applied to this problem [29], metaheuristic search remains largely unexplored in this setting. Third, the study provides evidence from a Latin American public hospital, a setting largely absent from the current evidence base. The remainder of the paper reviews related work (Section 2), describes the data and methods (Section 3), reports and discusses the results (Section 4 and Section 5), and concludes (Section 6).

## 2. Related Work on Machine Learning for Critical Care Readmission Prediction

ML models have been applied to predict unplanned readmission to critical care, outperforming traditional scoring systems with pooled sensitivities of 0.711 against 0.607 and specificities of 0.899 against 0.699 [30]. The advantage is nonetheless bounded. Using data from more than 400 intensive care units, separate models for post-discharge death and for early readmission reached areas under the receiver operating characteristic curve (AUC) of 0.92 and 0.71, respectively [31], suggesting that the ceiling lies in the outcome itself rather than in the volume of data available. Some models have reached the workflow itself. Routinely collected records predicted inpatient and critical care admission at each hour of an emergency department stay, and the models ran on live data [32].

Tree-based methods dominate this literature, appearing in 53% of studies, followed by neural networks (33%), regularized logistic regression (28%), and support vector machines (23%), with a median AUC of 0.68 [33]. In critical care, gradient boosting applied to electronic health record data reached an AUC of 0.76 [34], while XGBoost combined with Bayesian optimization and Shapley additive explanations (SHAP) reached 0.92 on data from the Medical Information Mart for Intensive Care (MIMIC) database [29]. Comparisons across algorithm families show a modest advantage for deep learning over logistic regression, with a mean AUC difference of 0.06, and no significant differences for tree-based and kernel-based methods [35], suggesting that performance gains depend less on the model family than on the information available to it. A third line of work replaces population-wide models with patient-specific ones. These identify the Markov blanket of the outcome for the case at hand and average predictions over the resulting structures under a Bayesian criterion [36]. The models developed here are population-wide.

The predictors driving these models are largely physiological. Vital signs and laboratory values recorded near discharge, along with mechanical ventilation and age, consistently rank among the most influential features [30]. Richer modalities perform better still: on MIMIC-III data, physicians’ discharge notes outperformed every other source, including structured records and multivariate time-series, for predicting unplanned readmission [37]. Nursing information appears less frequently; however, incorporating nursing assessments into routine clinical data improved early readmission prediction among high-risk discharges [38].

Despite this growing body of evidence, three aspects remain comparatively unexplored. First, the strongest-performing models rely on data modalities that are rarely available in routine registries at the time of transfer, such as continuous physiological signals, laboratory panels, and free-text notes, which limits their use in supporting the discharge decision itself. Second, hyperparameter optimization in this literature has relied on conventional strategies, most notably Bayesian techniques applied to XGBoost [29]; whether metaheuristic search offers advantages for the mixed, high-dimensional configuration spaces these models involve remains an open question. Third, the evidence rests heavily on two North American research databases, MIMIC and the eICU Collaborative Research Database, so transportability to other registries and health systems remains unestablished, and Latin American public hospitals are largely absent. These gaps are compounded by methodological concerns: in the most recent meta-analysis, every included study was rated at high risk of bias, and external validation was scarce [30]. This study addresses these concerns through nested stratified cross-validation with preprocessing embedded in the training folds, explicit reporting of the hyperparameter search spaces and optimizer settings, and reporting in line with the TRIPOD + AI guideline [39], while acknowledging that external validation remains a necessary next step.

## 3. Methods

### 3.1. Study Setting and Data

This study uses data from a two-phase cohort study conducted in the adult critical care unit of Hospital Dr. Gustavo Fricke, a 560-bed public high-complexity hospital in Viña del Mar, Chile, which ranks sixth in complexity nationally, serves a catchment population of approximately 1.1 million inhabitants, and offers all specialties except neurosurgery. The unit is a mixed medical-surgical UPC and comprises 42 beds: 16 in intensive care and 26 in intermediate care. This configuration is the standard model in the Chilean public system, where medical and surgical critically ill patients are managed together within the UPC [1], and dedicated medical ICUs are not the norm. It admits adult medical and surgical patients from the emergency department, post-anesthesia recovery unit, hospital wards, and other healthcare facilities, except neurosurgical patients, who are referred to another hospital, and complex cardiac patients, who are managed in the same hospital’s separate cardiovascular critical care unit. Between September 2024 and February 2025, 568 patients were admitted to the unit, corresponding to approximately 1100 admissions per year. Bed occupancy is not routinely reported by the unit; during the study period, occupancy on the day of transfer exceeded 80% in 83.3% of the recorded transfers.

Intensive and intermediate care beds are located on the same floor and share medical and nursing staff, who rotate between both levels every four months, as well as the medical and nursing heads of the unit. Patients admitted to intensive care are usually stepped down to intermediate care once their need for organ support decreases, and are transferred to a general ward from there. The nurse-to-patient ratio is 1:2 to 1:3 in intensive care, reaching 1:1 for the most complex patients, and 1:6 in intermediate care.

In Chile, adult intensive care medicine is a recognized medical specialty, certified by universities or by the National Corporation for the Certification of Medical Specialties (CONACEM). The national guideline requires this specialty for the medical head of the UPC and for the technical heads of its intensive and intermediate care areas, whereas resident physicians must hold a primary specialty in internal medicine, surgery, anesthesiology, or emergency medicine and training in intensive care medicine. For nurses, postgraduate critical care programs are available but are not required for clinical positions, which require prior experience in the care of critically ill patients; university training in critical care is required for supervisory positions [1].

Transfer decisions are made in a joint round held from Monday to Friday by the unit’s intensivists, including the heads, coordinators, physicians on duty, and fellows, in which each patient’s clinical course is reviewed. Patients are first stepped down from intensive to intermediate care, and the same process is used to decide transfer from intermediate care to a general ward. These decisions rely on clinical judgment, without a standardized discharge score or checklist. The coordinating physician and nurse then present candidate patients at a bed-management meeting with the hospital directorate, where beds in the receiving wards are assigned. Because these beds must first be vacated, authorized transfers frequently take place in the afternoon or at night; during the study period, 39.4% of transfers occurred at night (between 20:01 and 07:59) and 18.3% on weekends or holidays. Bed pressure also affects step-down within the unit, as patients may occasionally be moved from intensive to intermediate care to free a bed, even when still clinically vulnerable.

Patients were eligible if they were 18 years or older, had remained in the unit for at least 24 hours, and were transferred to a general ward. Patients who died in the unit, who were under therapeutic limitation orders or receiving palliative care, and those discharged home or to another healthcare facility were excluded.

Data were collected in two phases. The first was prospective and ran from 1 September 2024 to 28 February 2025. We identified eligible patients from the daily census and shift handover records, and extracted their data from the nursing sheet and clinical record, consulting the treating nursing staff when an entry was ambiguous. The second phase was retrospective and reviewed an anonymized list of patients hospitalized in the unit between 1 January and 31 August 2024, from which we retrieved only readmitted patients. For each included patient, we consulted the hospital information system to obtain the discharge date and discharge condition.

We defined early readmission as an unplanned return to the UPC, to either an intensive or an intermediate care bed, within seven days of transfer to a general ward during the same hospitalization. Changes in care level within the UPC during the index stay, including escalation from intermediate to intensive care, were not counted as readmissions, because the patient had not been transferred out of the unit. All predictors refer to the index UPC stay: length of stay before UPC admission and days in the UPC were computed from the hospital admission, UPC admission, and first UPC discharge dates of the index episode, and we used no information from the readmission episode. We assigned patients without comorbidities a comorbidity count of zero.

The final analytical sample comprised 371 patients, of whom 41 were readmitted. This proportion does not reflect the unit’s early readmission incidence because the retrospective phase included only readmitted patients; in the prospective phase, 18 of 348 patients were readmitted (5.2%). As the unit does not keep routine readmission statistics, this figure is the best available estimate of its natural early readmission rate. The design is therefore case-enriched. Case enrichment was adopted because the prospective phase yielded only 18 readmissions, too few to develop multivariable models, and it more than doubled the number of events available. Retrospective cases were identified in the same unit, under the same eligibility criteria and outcome definition, and their data were extracted from the same sources. We compared all variables between readmitted patients in the two phases; they differed only in the presence of peripheral and central venous catheters at transfer, supporting pooling for model development. In addition, the monthly number of readmissions was similar in both periods (2.9 in the retrospective phase and 3.0 in the prospective phase), suggesting no major change in readmission incidence over time. Provided that the retrospective cases are representative of all readmitted patients, this enrichment leaves sensitivity, specificity, and discrimination largely unaffected, but it shifts calibration and prevalence-dependent measures: predicted probabilities, precision, and the Brier score refer to the analytical prevalence (11.1%) rather than the observed incidence (5.2%), and clinical deployment would require recalibrating the models to the target prevalence [40,41].

The sample size was determined by the duration of the prospective phase, as no prior estimate of the unit’s readmission rate was available for a formal calculation. With 41 events and 13 predictors, the study had about three events per predictor, below the commonly recommended minimum of ten, so the sample supports only an exploratory analysis.

The Scientific Ethics Committee of Nursing (CECENF), Faculty of Nursing, Universidad Andrés Bello, approved the study protocol on 17 July 2024 (record No. L4/CECENF/03-2024), and the hospital directorate authorized the study. Patients included in the prospective phase provided written informed consent. For the retrospective phase, which reviewed only anonymized records of patients already discharged from the unit, the committee waived this requirement.

### 3.2. Data Preprocessing and Feature Engineering

The analytical dataset contained 13 predictors. Four were numerical: age, length of stay before UPC admission, days in the UPC, and number of comorbidities. Nine were categorical: care level at UPC admission (intensive or intermediate), sex, admission diagnosis (nine categories), unit of origin, and five comorbidity fields, each recording the coded type of one comorbidity in the order in which it was entered (14 possible types, or “none” when fewer comorbidities were recorded). Categorical predictors had between 2 and 18 levels. We used the unit of origin as recorded: 13 records carried free-text annotations to the six protocol codes and were treated as distinct categories. All 13 predictors were fully observed for the 371 patients; absent comorbidities were coded as zero in the comorbidity count and as “none” in the comorbidity fields, so no imputation was required. We shuffled records with a fixed seed before partitioning.

We transformed categorical predictors with target encoding, replacing each category with an estimate of the outcome rate within that category [42]. As implemented in scikit-learn, the estimate is shrunk toward the overall prevalence with an empirical-Bayes smoothing factor, and we encode training observations via five-fold cross-fitting so that no observation is encoded using its own outcome. This representation keeps dimensionality low relative to one-hot encoding, which is relevant for a sample of this size. Numerical predictors were rescaled to the unit interval with min–max normalization, which prevents variables with larger ranges from exerting a disproportionate influence on distance- and margin-based learners such as support vector machines [43]; although tree-based models are insensitive to feature scale, the same preprocessing was applied to all models for consistency.

### 3.3. Nested Cross-Validation Setting

Model development used a stratified nested cross-validation setting with five outer folds and four inner folds as shown in Figure 1. Stratified partitions were used to preserve approximately the outcome class distribution in the dataset. In each outer iteration, one fold was held out for evaluation, while the remaining four folds formed the outer training set. This training set was subsequently divided into four inner folds for hyperparameter optimization.

Nested cross-validation was used to reduce the optimistic bias associated with hyperparameter optimization by separating model selection from performance evaluation. Hyperparameters were selected within the inner loop, using either Bayesian optimization or the genetic algorithm, whereas the outer folds evaluated the complete selection and training procedure using observations excluded from optimization [44].

Performance was summarized using the mean and standard deviation across the five outer test folds. The standard deviations describe variation across partitions and should not be interpreted as confidence intervals, because the corresponding training sets overlap. Model rankings were therefore interpreted descriptively, and small differences were not regarded as evidence of superior predictive performance.

To prevent information leakage, we embedded the transformation of the variables in the following way: we estimated the encoding and scaling parameters on the training folds only and then applied them to the corresponding validation fold, ensuring that no information is shared among the training, testing, and validation sets [45,46].

### 3.4. Machine Learning Models

We employed five supervised machine learning algorithms to predict patients’ risk of readmission: logistic regression (LR), a support vector machine classifier (SVC), a decision tree (DT), a random forest (RF), and an extreme gradient boosting model (XGBoost). These algorithms represent distinct learning paradigms, ranging from conventional linear classification and margin-based learning to individual tree-based methods and ensemble approaches based on bagging and boosting. Their inclusion enabled the evaluation of models with different levels of complexity, interpretability, and capacity to capture nonlinear relationships and interactions among clinical predictors.

The class imbalance problem was addressed through class weighting during the model fitting procedure: fixed for SVC and LR, hyperparameter-tuned for RF and DT, and sample-weighted for GBM. No resampling technique was used due to the sample size of this study. All models were implemented in Python using the scikit-learn package version 1.7.1 and the xgboost package version 3.4.1. [47].

### 3.5. Hyperparameter Optimization

A key aspect of machine learning algorithms is their dependence on hyperparameter configuration, as performance depends on how these variables are specified. Table 1 reports the range of hyperparameters selected for the model specification and optimization process. The selection of the range of values is based on common practices for each model. In this sense, researchers have proposed a wide range of optimization strategies, each with different search and optimization mechanisms and trade-offs between computational cost and efficiency. Conventional methods, such as grid search or random search, rely on searching configurations within a space defined by the analyst before the fitting procedure [48].

Recent hyperparameter optimization methods have been proposed; in particular, Bayesian Optimization has gained attention for its ability to specify hyperparameter configurations based on sequential evaluations. Alternatively, other methods rely on metaheuristic optimization processes, including genetic algorithms, which perform adaptive searches over complex, high-dimensional, mixed-type hyperparameter spaces [48,49].

In this study, two hyperparameter optimization strategies were evaluated and compared: Bayesian optimization and a genetic algorithm, both maximizing the same objective function, f(λ)=Precision(λ), defined as the precision of the readmission class at the default classification threshold, averaged over the four inner validation folds under identical fold partitions. We chose precision as the optimization target because it corresponds to the positive predictive value: in a unit operating at high occupancy, each false alarm may delay the transfer of a stable patient and occupy a bed needed by another critically ill patient. Because precision depends on outcome prevalence, the configurations selected at the analytical prevalence of 11.1% may not be optimal at the natural prevalence of about 5%; we therefore also report the AUC, which does not depend on prevalence, and the Brier score, a proper scoring rule.

Because optimizing precision in isolation can favor conservative classifiers that flag very few patients, we summarized the selected configurations with precision and F1-score computed over both classes, together with accuracy, AUC, and the Brier score. This selection reflects each method’s optimization strategy. Bayesian optimization balances exploration and exploitation while remaining sample efficient compared to traditional search methods [50]. Genetic algorithms preserve strong configurations across generations, combine useful components from different solutions through crossover, escape local regions, and capture complex interactions while handling mixed hyperparameter types [48]. Furthermore, traditional grid search becomes inefficient as the number of selected hyperparameters increases, as it tries every combination of the provided values. In contrast, random search samples configurations independently, risking leaving promising regions of the space unexplored [51].

### 3.6. Hyperparameter Optimization Strategies

#### 3.6.1. Bayesian Optimization

As noted above, we used a Bayesian optimization strategy to select hyperparameter configurations. Let λ∈Λ be the vector of parameters within a search space Λ. The objective is to maximize f(λ) as defined above.

The steps conducted are detailed below:

1. **Initial evaluation:** A small set of initial hyperparameter configurations, λ∈Λ, is selected and evaluated on the validation data.
2. **Surrogate model estimation:** The resulting objective values are used to fit a probabilistic surrogate model, ($\hat{f}\left(\lambda \right)$), which approximates the relationship between each hyperparameter configuration and the objective while quantifying the uncertainty associated with its predictions.
3. **Selection of the next configuration:** At iteration *b*, an acquisition function, a(λ), is optimized to identify the next hyperparameter configuration that will be evaluated:
  (1) $$\underset{\lambda \in \Lambda }{\arg\max}\left\{a\left(\lambda \right)\right\}$$
  The acquisition function balances the exploration of uncertain regions of the search space with the exploitation of regions expected to produce favorable objective values.
4. **Model evaluation:** The selected configuration, $\lambda_{b+1}$, is used to train the model with the training data and is evaluated on the validation set, which yields a new value of the objective on the validation folds.
5. **Surrogate model update:** The surrogate model is updated to improve its approximation of the objective function.
6. **Iterative search:** Steps 3–5 are repeated until the maximum number of iterations is completed.
7. **Selection of the optimal configuration:** Once the optimization process is complete, the hyperparameter configuration with the best observed objective values is selected and subsequently used to estimate the final model.

#### 3.6.2. Genetic Algorithm

Genetic algorithms are population-based evolutionary metaheuristics that improve a set of candidate solutions through selection, recombination, and mutation operators inspired by natural evolution. Each individual encodes a hyperparameter configuration $\lambda =(\lambda_{1},\ldots ,\lambda_{p})\in \Lambda$, and its fitness is f(λ), the positive-class precision averaged over the four inner validation folds defined above. The steps are detailed below:

1. **Initial population:** The algorithm was implemented so that each individual encodes a hyperparameter configuration, $\lambda =(\lambda_{1},\ldots ,\lambda_{p})\in \Lambda$. The initial population consists of N=5 individuals, sampled without replacement from a discretized grid of the search space.
2. **Evaluation Fitness:** The fitness of each individual is the objective f(λ) averaged across the four inner cross-validation folds, with higher precision indicating better fitness. We used the same objective function and the same total number of model evaluations for Bayesian optimization, so performance differences reflect the search strategy rather than the optimization target or budget.
3. **Iterative process:** This is repeated for G=500 generations, with the following steps performed at each iteration:
  - **Crossover:** Each parent is selected by tournament selection: three individuals are drawn from the current population, which includes the elite individual carried over unchanged from the previous generation, and the fittest is chosen; the two parents are drawn independently. For each hyperparameter *j*, the child is generated according to Equation (2). This process is repeated until $n_{children}$ offspring are generated.
    (2) $$\lambda_{j}^{child}=\alpha \lambda_{j}^{\left(1\right)}+\left(1-\alpha \right)\lambda_{j}^{\left(2\right)},\;\alpha ∼U\left(0,1\right)$$
    Integer-valued hyperparameters are rounded down; categorical hyperparameters are not blended, and the offspring receive a default category that can only change through mutation. Four offspring are generated per generation.
  - **Mutation:** Each hyperparameter of each offspring mutates independently with a probability of $p_{m}=0.30$ (0.40 for the SVC): numerical genes according to Equation (3), where clip(·) enforces the feasible bounds, and categorical genes by uniform resampling from their admissible values.
    (3) $$\lambda_{j}\leftarrow \mathrm{clip}\left(\lambda_{j}\cdot u\right),\;u∼U\left(0.1,2.0\right)$$
4. **Algorithm output:** The algorithm outputs the hyperparameter configuration with the best fitness according to Equation (4).
  (4) $$\underset{\lambda \in P}{arg max}f\left(\lambda \right)$$

### 3.7. Evaluation Criteria

As early readmission was the minority class, we assessed model performance using several complementary metrics: accuracy, precision, F1-score, area under the receiver operating characteristic curve (ROC-AUC), Brier score, and Recall for the positive class, this is, for patients with readmission in our data. Multiple metrics are particularly important in imbalanced classification, as no single measure fully captures predictive performance [52]. Accuracy quantifies the overall proportion of correctly classified patients but may overstate performance when the majority class predominates. Precision measures the proportion of patients predicted to be readmitted who were actually readmitted, thereby reflecting the reliability of positive predictions. The F1-score denotes the harmonic mean of precision and recall and, consequently, evaluates the balance between the reliability of positive predictions and the model’s ability to identify readmitted patients. ROC-AUC assesses the model’s capacity to discriminate between readmitted and non-readmitted patients across all possible classification thresholds. Finally, the Brier score measures the mean squared difference between predicted probabilities and observed outcomes, assessing the overall accuracy of the probabilistic predictions; lower values indicate better performance [53,54].

### 3.8. Model Explanation

To measure how much each feature contributes to building the model’s nodes, we used the impurity-based feature importance of the random forest configuration selected by the genetic algorithm, refitted in each of the five training folds, and reported the mean and standard deviation across folds. For a tree-based model, each node contains a mixture of observations from the outcome classes. This mixture is measured through the Gini impurity index I(t), which involves the proportion $p_{k,t}$ of observations from class *k* at node *t*. For a binary classification problem, a pure node containing only one class has I(t)=0, while a node with a 50:50 distribution has the maximum binary Gini impurity I(t)=0.5.

Feature *j*’s importance in a tree is the sum of the weighted impurity decreases over all nodes where that feature is used. For models that use more than one tree, such as random forests, importance is the average across its *M* trees [55]. Impurity-based importance is known to be biased toward continuous and high-cardinality predictors [55]. Because target encoding converts categorical variables into outcome-informed numerical ones, we interpret the importance of encoded features, such as unit of origin, admission diagnosis, and the comorbidity fields, with caution [56].

#### Use of Generative AI

During the preparation of this manuscript, the authors used ChatGPT to assist with English-language editing and structural refinement.

## 4. Results

### 4.1. Descriptive Results

The database comprised 371 patients from the UPC. Table 2 shows the distribution of the data by admission and readmission status, along with demographic and clinical characteristics. The dataset contains 41 readmitted patients (11.1%) and 330 non-readmitted patients (88.9%), indicating a marked class imbalance. As noted in Section 3, this proportion reflects the case-enriched design rather than the unit’s incidence. Because the analytical sample was enriched with readmitted cases, the proportions in Table 2 describe its composition and should not be interpreted as natural readmission rates; we therefore compare the distribution of each characteristic between readmitted and non-readmitted patients. Readmitted patients were more often men (58.5% versus 51.8%) and more often admitted to intensive care (51.2% versus 32.1%). Gastrointestinal diagnoses accounted for nearly half of readmitted patients (48.8% versus 23.9% of non-readmitted patients), whereas cardiovascular diagnoses were less frequent among them (9.8% versus 20.9%).

Figure 2 shows the distribution of numerical features considering the readmission category. For patient age, the distributions overlap substantially, with readmitted patients showing a higher median age and a narrower interquartile range. For the number of days in the UPC, readmitted patients have a higher median stay and a wider distribution, suggesting that longer stays in the critical care unit may be associated with readmission, potentially reflecting greater illness severity or clinical complexity. The length of stay before UPC shows that most non-readmitted patients spent approximately 0 to 1 day in the hospital before entering the UPC, whereas the readmitted group shows substantially greater variability. Finally, both groups have a median of about two comorbidities. However, the readmitted group is more concentrated between two and three, while the non-readmitted group includes patients with zero or one, suggesting a slightly greater comorbidity burden among readmitted patients.

### 4.2. Model Optimization

Figure 3 shows, for each learner, the fitness of the elite individual (positive-class precision averaged over the four inner folds) across the 500 generations of the genetic algorithm, together with the accuracy of the same individual for context. Figures display the mean of the metrics across the nested cross-validation setting. The largest improvements were observed for the ensemble models, particularly the random forest, which achieved the highest positive-class precision while maintaining an accuracy close to 0.90, i.e., close to the majority-class proportion. Gradient boosting also performed strongly, showing rapid improvement in the initial iterations and smaller gains toward the end of the optimization process. In contrast, the decision tree reached a comparatively lower performance plateau, while logistic regression and the support vector classifier showed limited improvement after their initial iterations. These findings suggest that the larger hyperparameter spaces of the ensemble models provided greater opportunities for evolutionary optimization. Nevertheless, because the non-readmission class was the majority class, we interpreted accuracy relative to the majority class baseline and considered it alongside precision and other minority-class metrics.

### 4.3. Model Performance

Table 3 shows the results of the Bayesian optimization process for hyperparameter optimization in the nested cross-validation setting. Under nested cross-validation with Bayesian hyperparameter optimization, RF achieved the highest mean accuracy (70.8%, SD 3.01%), closely followed by XGboost (70.6%, SD 8.96%). LR yielded the highest mean AUC (71.7%, SD 3.96%), followed by RF (70.2%, SD 5.84%), indicating that the accuracy ranking differed from the discrimination ranking. LR also obtained the highest mean precision (52.1%, SD 3.01%), whereas RF achieved the highest recall (55.2%, SD 15.6%). Mean F1-scores were similar across models, ranging from 47.0% for DT to 50.6% for XGBoost. SVC recorded the lowest reported Brier score (10.7, SD 0.67), followed by RF (13.9, SD 2.15).

Variability across the outer folds differed by metric and model. RF showed relatively consistent accuracy, whereas XGBoost exhibited greater variation. Recall varied considerably for RF and XGBoost, with standard deviations of 15.6 and 15.4 percentage points, respectively. DT had the lowest mean accuracy and AUC, with its AUC of 53.3% indicating discrimination close to chance on average. Overall, no model led across all metrics: LR provided the highest mean discrimination, RF combined comparatively high accuracy and recall, and SVC achieved the lowest probability prediction error according to the reported Brier scores.

The hyperparameter configurations selected through inner cross-validation within each of the five outer folds are reported in Supplementary Table S1. Selected configurations varied across folds, particularly in tree depth and ensemble size. The sigmoid kernel was selected for SVC in four folds, whereas LR showed relatively similar values of *C* across folds.

Table 4 shows model performance under the specifications identified by the genetic algorithm in the nested cross-validation setting. Under nested cross-validation with genetic algorithm hyperparameter optimization, XGBoost achieved the highest mean accuracy (85.7%, SD 16.6%), whereas LR obtained the highest mean AUC (75.2%, SD 1.42%), closely followed by XGBoost (75.0%, SD 19.2%) and RF (73.3%, SD 1.54%). RF achieved the highest mean precision (61.7%), F1-score (61.3%), and recall (61.3%), indicating a comparatively favorable balance between identifying positive cases and limiting false-positive predictions. These results suggest that model rankings depended on the evaluation metric, with no single model outperforming the others across all measures.

Performance variability also differed across models. LR and RF showed relatively consistent AUC values across the outer folds, while XGBoost exhibited substantially greater variability in both AUC and accuracy. RF’s recall and F1-score also varied considerably, with standard deviations of 19.3 and 16.9 percentage points, respectively. SVC yielded the lowest mean Brier score (0.10, SD 0.01), despite having the lowest AUC (61.4%), illustrating that probability prediction error and discrimination did not produce the same model ranking.

The results of the genetic algorithm in the nested cross-validation setting are shown in Table 5, serving as a comparative and consistent result of the algorithm in the inner-loop setting. The results are used as a robustness check and correspond to the performance on the validation sets shown in Figure 1. The tuned random forest (RF) achieved the highest mean accuracy (89.43%) and precision (71.33%). Its accuracy also varied within a narrow range (88.89–89.90%), suggesting relatively consistent performance across the evaluated folds. XGBoost ranked second for both metrics, with an accuracy of 85.45% and precision of 62.54%; however, its wider ranges—particularly for accuracy (75.41–89.22%)—indicate greater sensitivity to the data partition.

The decision tree achieved an accuracy of 82.85%, but its precision was lower at 52.71%, indicating a larger proportion of false positives among its positive predictions. Logistic regression and SVC had the lowest mean accuracies, at 74.45% and 68.51%, respectively. SVC also showed substantial variation in both accuracy and precision across folds. Overall, these results descriptively favor RF for the two reported metrics. However, the minimum–maximum ranges describe observed variation, not confidence intervals, and they do not establish statistically significant differences between models. Recall and class prevalence would also be needed to assess how effectively these models identify positive cases.

The calibration plots are shown in Figure 4 for the RF and XGBoost models. Both models showed deviations from ideal calibration in the lower and intermediate probability groups, where predicted probabilities exceeded the observed positive proportions. Agreement was closer in the highest-probability group: mean predictions of approximately 0.56 corresponded to observed proportions of approximately 0.53 for Random Forest and 0.55 for XGBoost. This pattern suggests that positive outcomes were concentrated among observations assigned higher probabilities, while probabilities in the remaining groups were overestimated. For reference, a null model assigning the analytical prevalence (11.1%) to every patient has a Brier score of 9.8%. All models had Brier scores at or above this value (Brier skill scores between −0.02 for SVC and −1.62 for DT), indicating that their predicted probabilities were not more accurate than the prevalence alone. This is consistent with the overestimation observed in the calibration plots and with class weighting, which shifts predicted probabilities upward; predicted probabilities should therefore not be interpreted as absolute risks.

### 4.4. Feature Importance

Figure 5 presents the mean impurity-based feature importance for the random forest model under genetic algorithm optimization. Days in the UPC and patient age had the highest mean importance values, with comparatively limited variation across folds. Unit of origin, length of stay before UPC admission, diagnosis at admission, and the third comorbidity field also ranked relatively highly, although their importance values showed greater variability. The first and second comorbidity fields had intermediate importance, while care level at UPC admission, gender, total number of comorbidities, and the fourth and fifth comorbidity fields had lower values. These rankings describe the variables’ relative contributions to impurity reduction within the fitted models; they do not establish the direction or magnitude of associations with readmission, causal effects, or the predictive value of each variable in isolation.

## 5. Discussion

This proof-of-concept study developed and internally validated five machine-learning models for predicting unplanned readmission to the UPC within seven days of transfer to a lower-acuity ward. The analysis yields three main findings. First, the models showed moderate discriminatory capacity (AUC between 0.53 and 0.75), and no single model performed best across all metrics. Second, the effect of hyperparameter optimization was model-dependent: the genetic algorithm yielded higher AUC than Bayesian optimization for the tree-based models, most markedly for the decision tree and XGBoost, whereas Bayesian optimization performed better for the support vector classifier. Third, variables describing patients’ clinical trajectories, particularly days in the UPC, age, unit of origin, length of stay before UPC admission, and diagnosis at admission, made the greatest contribution to the random forest prediction according to impurity-based importance.

The discriminatory performance observed in this study is broadly consistent with previous research on critical care readmission. Under genetic algorithm optimization, the best-performing models achieved AUC values between approximately 0.70 and 0.75. These results are comparable to AUC values reported across machine-learning studies of hospital readmissions [57,58]. Although higher values have been reported for models developed using databases such as MIMIC and richer sources of laboratory, physiological, and textual information [59], the present models relied primarily on routinely collected clinical and nursing variables available at the time of transfer. In this sense, the results reinforce the statement that early critical care readmission is intrinsically difficult to predict, reflecting not only patients’ conditions at transfer but also subsequent care, organizational circumstances, and clinical deterioration that may not be observable at the time of prediction.

The comparison of optimization strategies suggests that the genetic algorithm may be beneficial for tree-based models: under nested cross-validation, it yielded higher AUC than Bayesian optimization for the decision tree (68.6% versus 53.3%), XGBoost (75.0% versus 60.5%), and random forest (73.3% versus 70.2%), whereas Bayesian optimization performed better for the support vector classifier (65.2% versus 61.4%). This pattern is consistent with population-based search being advantageous when the hyperparameter space contains several interacting, mixed-type dimensions. However, we ran each strategy once per outer fold, so we did not assess its stochastic variability, and XGBoost showed the greatest between-fold variability; these differences should therefore be regarded as exploratory. Logistic regression produced similar discrimination under both approaches (75.2% versus 71.7%), as its hyperparameter space is small (the regularization strength *C* and the mixing ratio) and offers fewer optimization opportunities.

Regarding feature importance, age and variables describing patients’ clinical trajectories ranked relatively highly in the random forest model. Age had one of the highest mean impurity-based importance values and comparatively limited variation across folds. This pattern is broadly consistent with studies reporting differences in readmission risk across age groups [60,61], although the present analysis does not identify the mechanisms underlying these differences. Days spent in the UPC and length of stay before UPC admission also had relatively high importance values. Hospitalization duration may capture aspects of illness severity or care complexity, but these interpretations cannot be established from feature importance alone, nor do the results indicate whether longer stays are associated with higher or lower predicted risk. Hospitalization-related variables have also been considered in previous supervised-learning studies of readmission [62,63].

Unit of origin and diagnosis at admission also ranked relatively highly, suggesting that the fitted model used information related to admission characteristics and care pathways. The importance of diagnosis at admission, represented through target encoding, is broadly consistent with its consideration in studies of readmission in specific clinical settings [64,65]. However, these rankings are specific to the fitted model, predictor set, and encoding choices. Impurity-based importance does not establish independent associations, causal effects, or the direction of readmission risk, and correlated predictors may share importance. The findings should therefore be interpreted as a description of how the model used the available variables rather than evidence of clinical mechanisms.

From a clinical perspective, the model should be regarded as decision support rather than a replacement for professional judgment. Once externally validated and recalibrated, a risk estimate generated at the time of transfer could complement existing clinical assessments by identifying patients who may benefit from reassessment, delayed transfer, closer ward monitoring, or follow-up by a critical care transition team; the present results do not yet support its use to guide transfer decisions. However, the appropriate classification threshold should reflect the consequences of different error types; for example, model thresholds can be aligned with the operating points of instruments already used in practice, so that gains in sensitivity or specificity are assessed under equivalent clinical conditions [66]. In this sense, model implementation should be guided not only by statistical performance but also by available capacity, clinical workload, and the relative costs of missed readmissions and unnecessary interventions [67]. Adoption also depends on fit with clinical workflows, information systems, and professional acceptance [68].

Furthermore, this study has several strengths. It provides evidence from a Latin American public hospital, a setting that is underrepresented in the critical care prediction literature, and uses variables that are routinely available when clinicians make the transfer decision. It also compares different learning paradigms and two hyperparameter optimization strategies using the same objective function, validation partitions, and evaluation criteria. Furthermore, the study incorporated processing operations, including target encoding and normalization, within a nested cross-validation setting to reduce information leakage, and assessed model performance using complementary threshold-based, discrimination, and probability-based metrics.

Despite these results, several limitations remain. The study was conducted in a single hospital with a moderate sample size and a small number of readmission events, limiting the statistical precision and transportability of the estimates. Given the low number of events per predictor, the selected hyperparameters, model rankings, and feature importance may be unstable. Combining prospective patients with retrospectively retrieved readmitted cases produced a case-enriched sample, which prevents direct interpretation of the predicted probabilities as risks under routine prevalence and may introduce selection and temporal bias, since the additional cases came from an earlier period in which discharge practices, bed pressure, or case mix may have differed. Such bias could affect discrimination, calibration, and threshold-dependent performance measures. Model performance could not be reliably estimated in the prospective cohort alone, as its 18 events would yield highly imprecise estimates; for an AUROC of 0.70, for instance, the 95% confidence interval would span approximately ±0.14. Recalibration to the natural prevalence must also be tailored to each modeling approach. For logistic regression, an intercept correction can account for outcome-dependent sampling [40], assuming that sampling depended only on the outcome and that the correction is based on the effective training prevalence, which differs from the analytical prevalence when class weighting is used. For tree-based models and support vector machines, predicted probabilities are not necessarily calibrated and are further distorted by class weighting, so an intercept shift is insufficient and prior-shift adjustments are valid only for already calibrated probabilities; these models would require logistic or isotonic recalibration in a sample with the natural prevalence, together with re-estimation of the target encodings. Furthermore, internal cross-validation does not replace temporal or external validation, and the models should be evaluated in new patients considering different time frames before clinical implementation. Moreover, the predictor set did not include physiological variables such as vital signs and their trajectories, laboratory measurements, treatment-related variables such as organ support or vasoactive drugs at transfer, nursing workload, staffing and bed occupancy at the time of transfer, or post-transfer factors such as ward care and follow-up, all of which may influence readmission risk and may explain part of the remaining predictive uncertainty. In addition, comorbidities were represented by five fields recorded in order of entry, so the same disease could appear in different predictors depending on documentation order, and free-text annotations to the unit of origin were kept as separate categories; both modeling choices may have produced sparse levels and unstable target encodings. Hyperparameters should also be optimized with prevalence-independent or clinically motivated objectives, such as the Brier score or net benefit at a clinically relevant threshold. Finally, impurity-based importance can favor continuous and high-cardinality variables and distribute importance among correlated predictors. We therefore interpret the reported importance values as predictive contributions to the specific model and setting rather than causal effects or evidence of associations.

Overall, the findings suggest the feasibility of predicting early UPC readmission using routinely collected information available at transfer and indicate that the choice of optimization strategy can affect model performance. The genetic algorithm yielded higher discrimination for most tree-based models, but this advantage must be weighed against between-fold variability, the prevalence dependence of the optimized metric, and the use of a single optimization run per strategy. Future research should externally and prospectively validate the models, recalibrate predicted probabilities to the natural readmission incidence, evaluate calibration and clinical utility, and determine whether integrating physiological, nursing, and organizational information produces improvements that are stable and clinically meaningful to support transfer decisions. Such studies should also represent comorbidities through disease-specific indicators or a validated comorbidity index and harmonize the unit of origin to clinically defined categories. In particular, retrieving all eligible patients from the retrospective period, including those not readmitted, would allow the models to be developed in the earlier period and validated temporally in the prospective cohort, providing both a natural-prevalence development sample and a genuine temporal validation.

## 6. Conclusions

This study suggests the feasibility of using routinely collected clinical and nursing information to predict unplanned readmission to a critical care unit within seven days of transfer. Across the five evaluated classifier models, performance depended on both the learning algorithm and the hyperparameter optimization strategy. The genetic algorithm was particularly beneficial for the tree-based ensemble models, although differences between models were small and no single model performed best across all metrics. Days spent in the UPC, age, unit of origin, length of stay before UPC admission, and diagnosis at admission ranked highest in impurity-based importance, although these rankings describe how the fitted model used the available variables rather than their clinical relevance.

These findings suggest that metaheuristic hyperparameter optimization may improve discrimination for models with complex and interactive hyperparameter spaces; however, this requires confirmation across multiple optimization runs. However, the results should be taken as proof of concept rather than evidence of immediate clinical readiness, as they were derived from a single-center, case-enriched sample with a limited number of readmission events. Before implementation, the selected model requires recalibration to the natural readmission prevalence, external and prospective validation, and assessment of clinically appropriate decision thresholds and net benefits depending on the context in which the model is used. Once validated, the model could complement professional judgment by identifying patients who may benefit from reassessment, enhanced monitoring, or transitional care following transfer from the UPC.

Overall, this study contributes to the growing evidence on data-driven decision support in critical care by suggesting that routinely available data, without highly specialized physiological or laboratory measurements, contain predictive information on early readmission, although its clinical usefulness remains to be established. Beyond predictive performance, the comparison between Bayesian and genetic optimization highlights the importance of considering the interaction between model architecture and hyperparameter search strategy when developing clinical prediction tools. From an operational perspective, incorporating readmission-risk estimates into the transfer process may support more informed allocation of scarce critical care resources while helping clinicians identify patients requiring closer surveillance after discharge. Nevertheless, the single-center design, moderate sample size, limited number of readmission events, and case-enriched analytical sample constrain the generalizability and calibration of the proposed models. Future research should therefore focus on multicenter and prospective validation using larger cohorts and natural readmission prevalence, together with model recalibration and the evaluation of clinically meaningful decision thresholds. Further studies should also examine whether incorporating longitudinal physiological measurements, laboratory data, nursing assessments, staffing and workload indicators, and post-transfer care information improves predictive performance and model stability. Finally, prospective implementation studies are needed to assess the models’ clinical utility, cost-effectiveness, interpretability, and impact on patient outcomes, readmission rates, and critical care resource utilization.

## Figures and Tables

**Figure 1:**
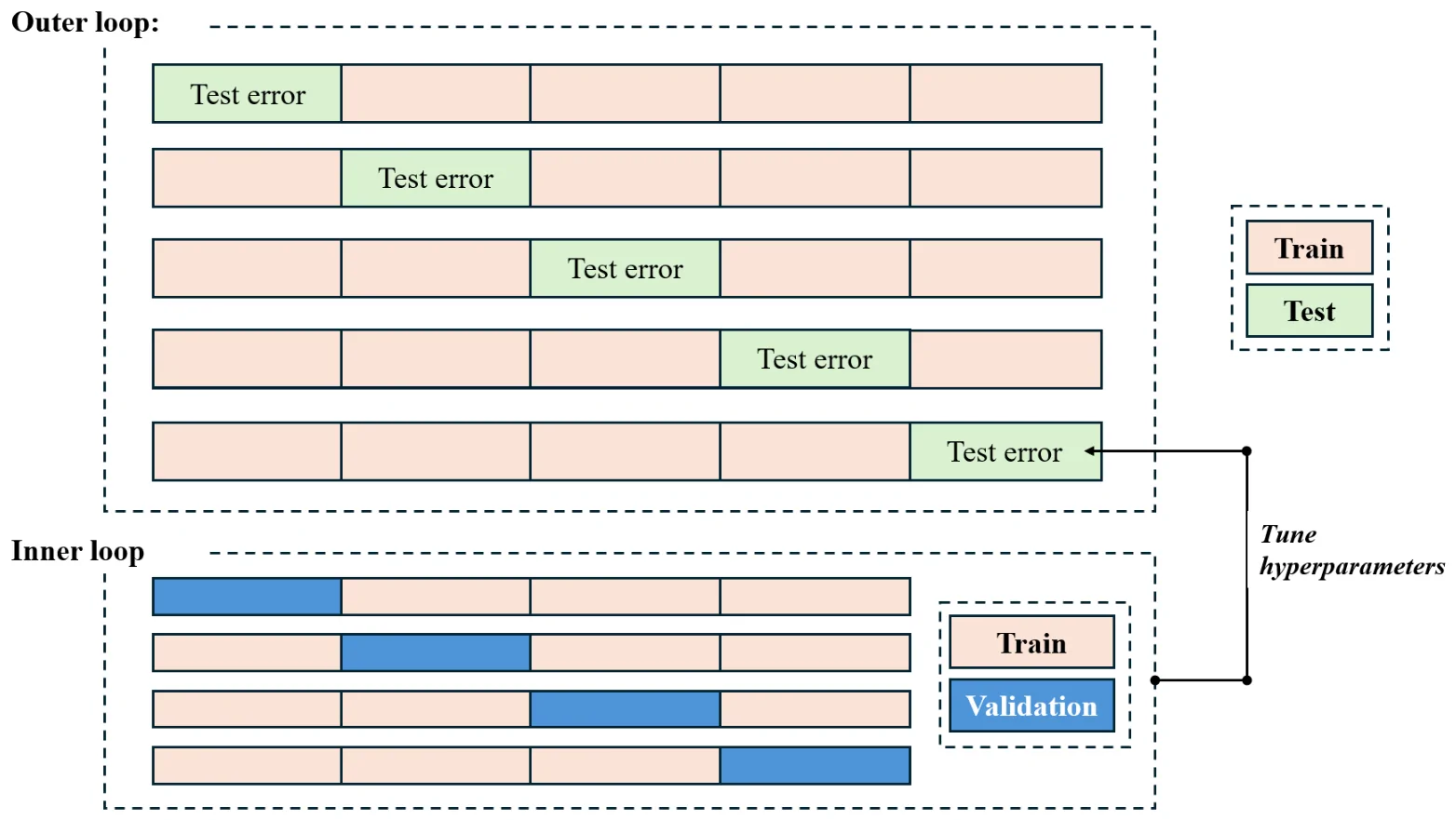
Nested cross-validation with five outer folds and four inner folds. In each outer iteration, hyperparameters are optimized through inner cross-validation using only the outer training data.

**Figure 2:**
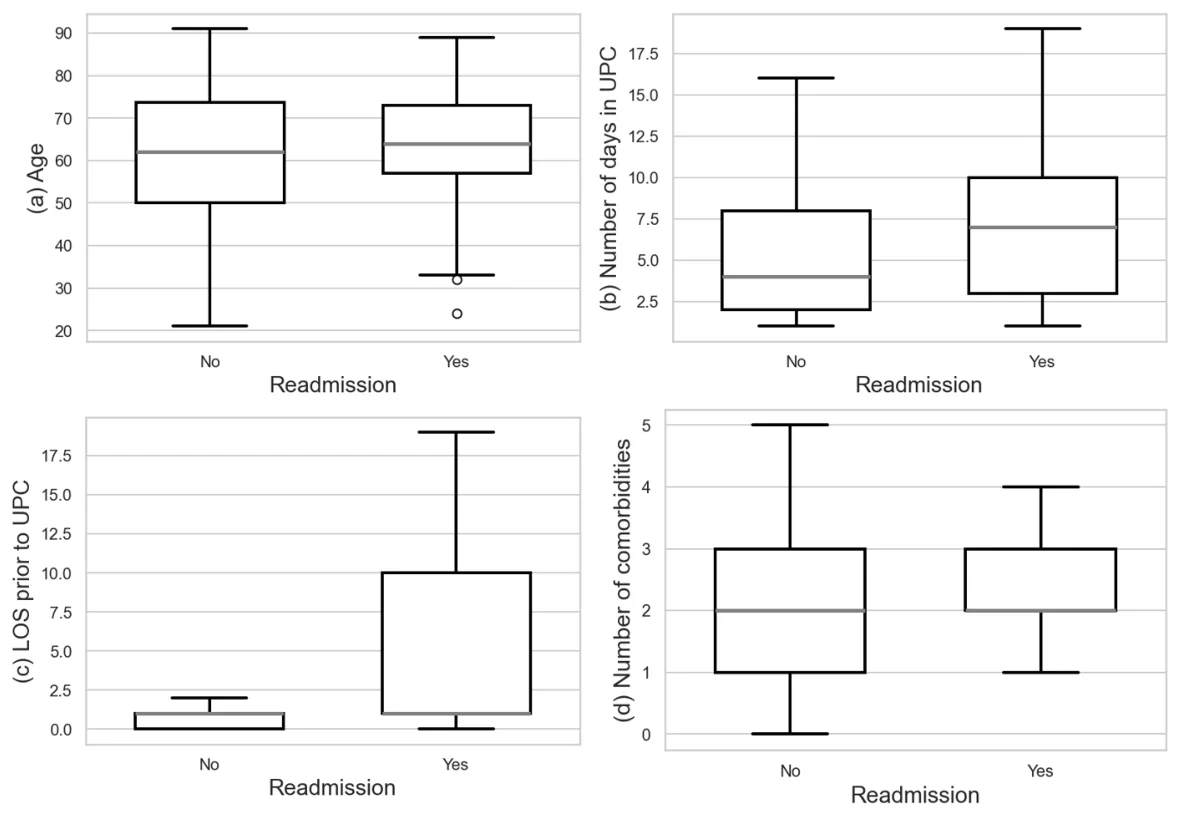
Distribution of numerical features of patients in the dataset considering the readmission status.

**Figure 3:**
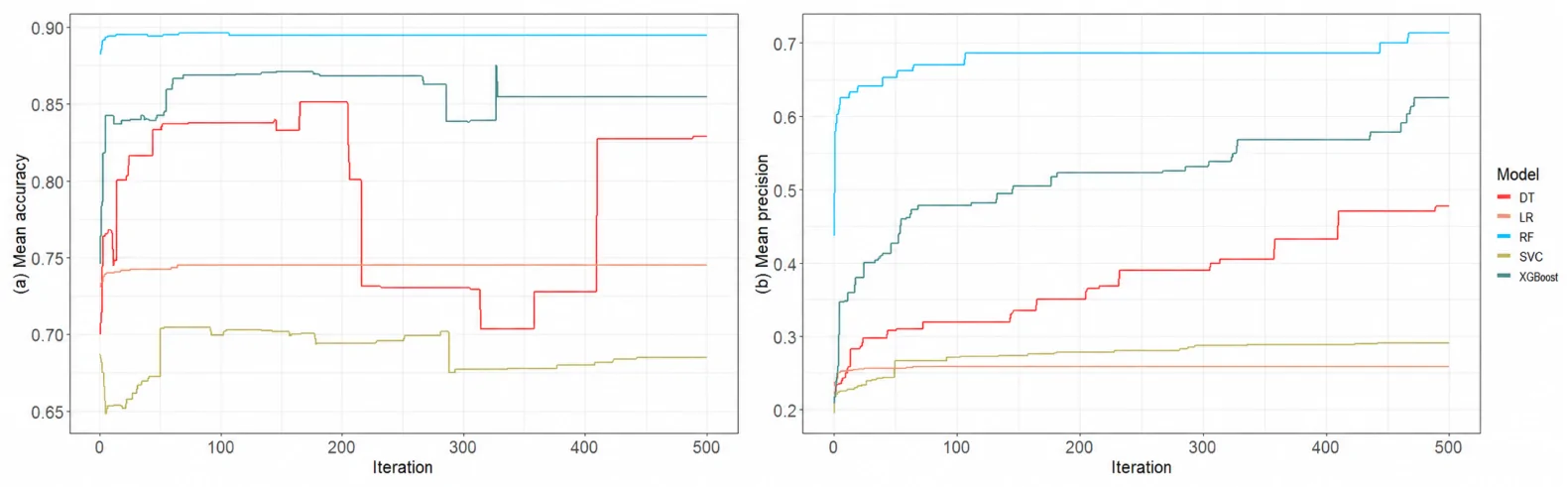
Evolution of the accuracy (**a**) and precision (**b**) through the iterations of the genetic algorithm for the models evaluated in the inner-cross-validation setting.

**Figure 4:**
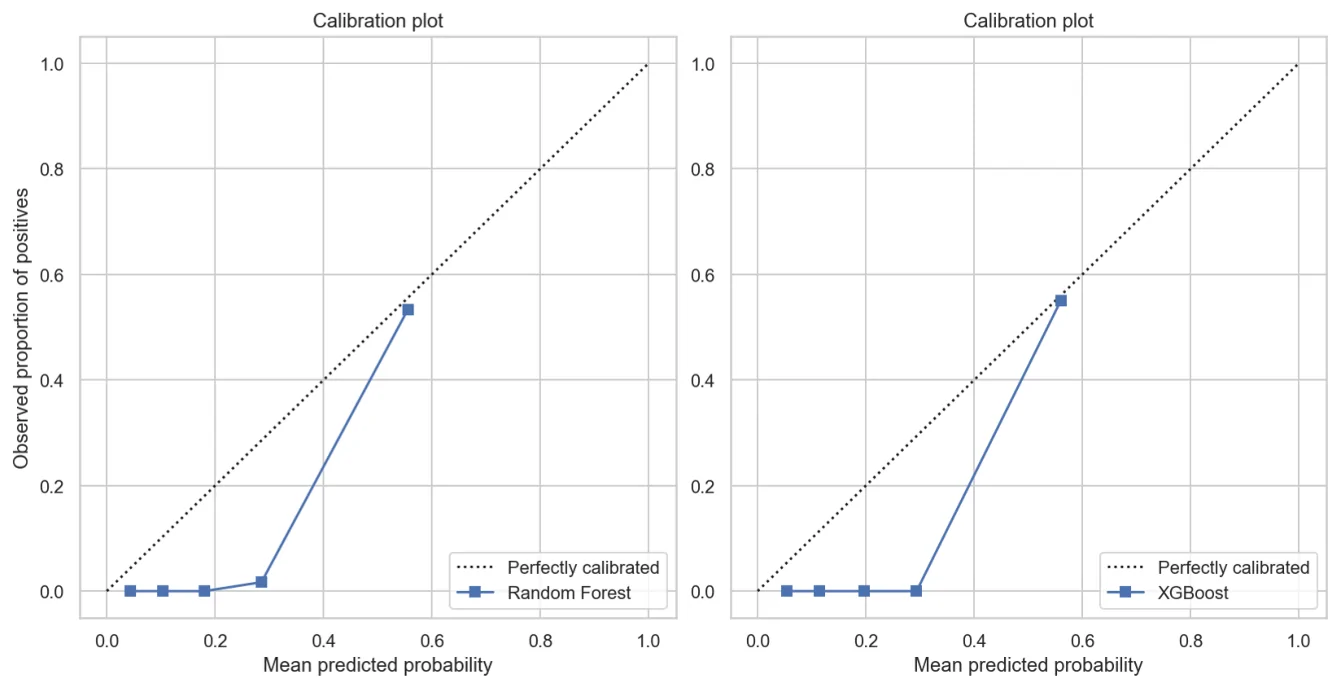
Calibration plots for RF (**left**) and XGBoost (**right**) models. Points compare mean predicted probabilities with observed positive proportions within probability bins. Diagonal line indicates perfect calibration. Points below and above the diagonal line indicate overestimation and underestimation, respectively.

**Figure 5:**
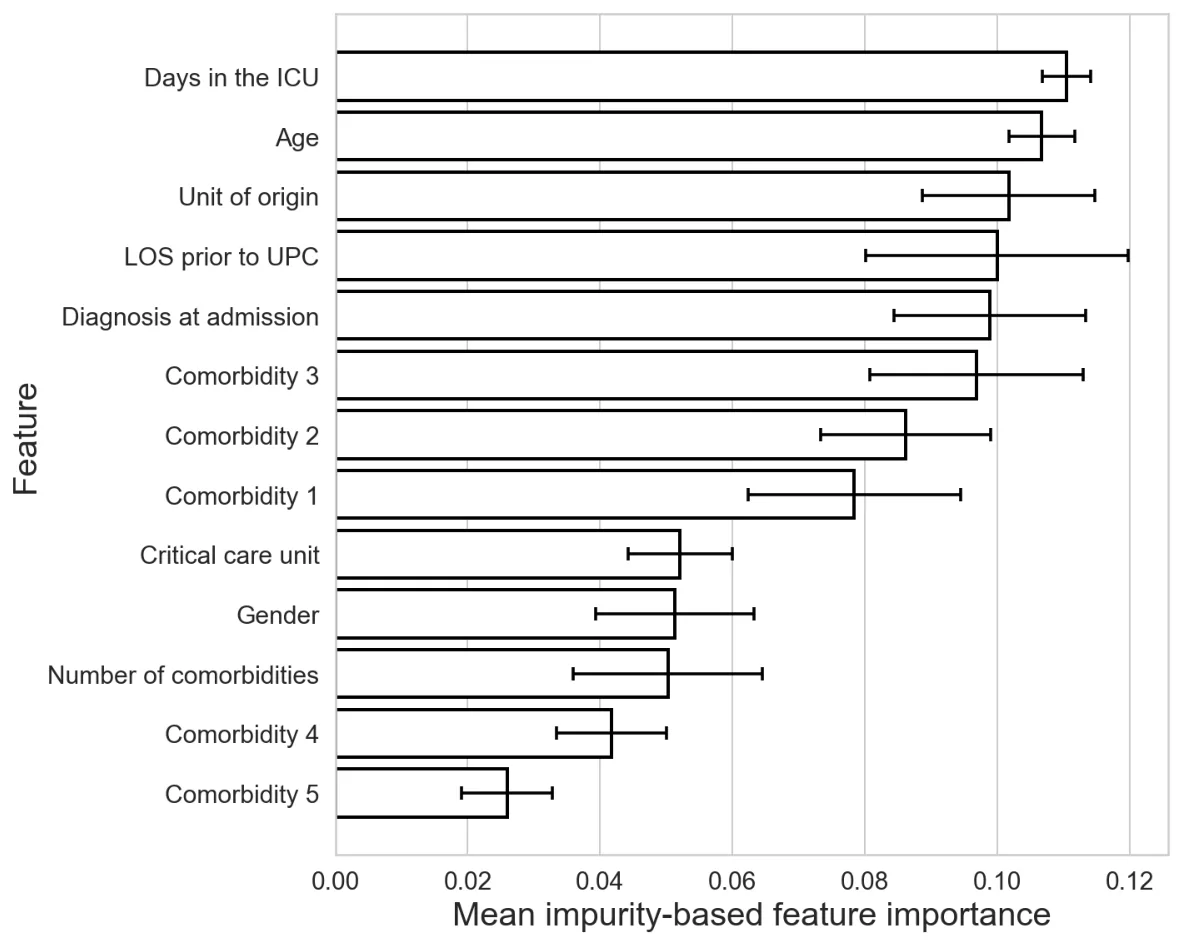
Feature importance for the random forest model across the cross-validation setting. Bars show the standard deviation of impurity importance across the different train–testing sets.

**Table 1:** Hyperparameter search spaces used for model optimization. Numerical intervals indicate the search bounds, while entries in braces list the candidate categorical values.

Model	Hyperparameter	Range
RF	Number of estimators	[2, 100]
	Criterion	{Gini, Entropy, Log-Loss}
	Max. depth	[1, 10]
	Min. samples to split	[2, 10]
	Min. samples per leaf	[1, 10]
	Max. features	{Square root, Logarithmic}
	Class weights	{None, Balanced}
XGBoost	Loss	{Log-loss, exponential}
	Learning rate	[0, 1]
	Number of estimators	[2, 100]
	Subsample	[0.001, 1]
	Min. samples to split	[2, 10]
	Min. samples per leaf	[1, 10]
	Max. depth	[1, 10]
	Class weights	{None, Balanced}
DT	Criterion	{Gini, Entropy, Log-Loss}
	Splitter	{Best, Random}
	Max. depth	[1, 10]
	Min. samples to split	[2, 10]
	Min. samples per leaf	[1, 10]
	Max. features	{Square root, Logarithmic}
	Class weights	{None, Balanced}
SVC	*C*	[0.00001, 10]
	Kernel	{Linear, RBF, Sigmoid}
	γ	[0.00001, 10]
LR	*C*	[0.00001, 10]
	Regularization-ratio	[0.00001, 1]

**Table 2:** Distribution of categorical features considering the subgroups of each variable, along with their shares considering the readmission status.

Feature	Group	Readmission	
		**No**	**Yes**
Gender	*Female*	159 (42.86%)	17 (4.58%)
	*Male*	171 (46.09%)	24 (6.46%)
Care level at UPC admission	*Intermediate care*	224 (60.38%)	20 (5.39%)
	*Intensive care*	106 (28.57%)	21 (5.66%)
Diagnosis at admission	*Cardiovascular*	69 (18.60%)	4 (1.08%)
	*Gynecological*	15 (4.04%)	0 (0.00%)
	*Respiratory*	67 (18.05%)	7 (1.89%)
	*Gastrointestinal*	79 (21.29%)	20 (5.39%)
	*Neurological*	21 (5.66%)	3 (0.80%)
	*Musculoskeletal*	13 (3.50%)	3 (0.80%)
	*Metabolic*	14 (3.77%)	1 (0.27%)
	*Renal*	34 (9.16%)	3 (0.81%)
	*Trauma*	18 (4.85%)	0 (0.00%)
Unit of origin	*Recovery*	116 (31.26%)	22 (5.92%)
	*Emergency*	164 (44.21%)	7 (1.88%)
	*Medicine*	16 (4.31%)	2 (0.54%)
	*Surgery*	7 (1.86%)	3 (0.81%)
	*Another hospital*	5 (1.35%)	1 (0.27%)
	*Other*	22 (5.93%)	6 (1.62%)

**Table 3:** Classification performance metrics for the models evaluated with the specification yielded by the Bayesian optimization process, considering the standard deviation (SD) due to the cross-validation setting. Accuracy, precision, F1-score, and recall metrics are measured in %. Standard deviations describe between-fold variability rather than confidence intervals. Metrics are calculated considering the positive class of the outcome variable.

Model	Accuracy (SD)	Precision (SD)	AUC (SD)	Brier (SD)	F1-Score (SD)	Recall (SD)
DT	54.3 (7.44)	47.2 (1.93)	53.3 (5.09)	25.8 (3.36)	47.0 (5.78)	49.7 (4.28)
LR	63.6 (6.95)	52.1 (3.01)	71.7 (3.96)	22.1 (4.16)	50.0 (0.01)	48.9 (10.1)
RF	70.8 (3.01)	51.5 (10.2)	70.2 (5.84)	13.9 (2.15)	50.3 (4.13)	55.2 (15.6)
SVC	62.4 (6.81)	48.9 (3.34)	65.2 (7.02)	10.7 (0.67)	49.8 (4.62)	48.0 (13.5)
XGBoost	70.6 (8.96)	49.1 (5.14)	60.5 (5.67)	20.6 (6.89)	50.6 (3.40)	51.0 (15.4)

**Table 4:** Classification performance metrics for the models evaluated in the nested cross-validation setting with the genetic algorithm. SD stands for standard deviation. Accuracy, precision, F1-score, and recall metrics are measured in %. Standard deviations describe between-fold variability rather than confidence intervals. Metrics are calculated considering the positive class of the outcome variable.

Model	Accuracy (SD)	Precision (SD)	AUC (SD)	Brier (SD)	F1-Score (SD)	Recall (SD)
DT	70.3 (9.55)	50.3 (7.00)	68.6 (3.94)	0.22 (0.01)	51.2 (3.93)	52.5 (3.97)
LR	65.7 (1.91)	54.5 (10.1)	75.2 (1.42)	0.22 (0.03)	51.2 (7.08)	49.8 (9.77)
RF	72.9 (8.45)	61.7 (14.3)	73.3 (1.54)	0.20 (0.01)	61.3 (16.9)	61.3 (19.3)
SVC	59.6 (4.34)	51.9 (14.1)	61.4 (3.59)	0.10 (0.01)	50.1 (12.8)	49.9 (15.6)
XGBoost	85.7 (16.6)	59.7 (9.43)	75.0 (19.2)	0.19 (0.30)	55.4 (14.4)	52.5 (17.4)

**Table 5:** Robustness assessment of genetic algorithm-based hyperparameter optimization across validation folds. Values represent mean accuracy and precision (%), with minimum and maximum values in parentheses. Metrics are calculated considering the positive class of the outcome variable.

Model	Accuracy (%)	Precision (%)
RF	89.43 (88.89–89.90)	71.33 (66.67–75.00)
XGBoost	85.45 (75.41–89.22)	62.54 (51.47–74.86)
DT	82.85 (68.18–87.20)	52.71 (39.22–52.65)
SVC	68.51 (56.67–72.40)	53.00 (25.01–59.86)
LR	74.45 (73.41–74.76)	55.00 (45.82–56.18)

## Data Availability

The data presented in this study are available on request from the corresponding author.

## Supplementary Materials

The following supporting information can be downloaded at https://www.mdpi.com/article/10.3390/jcm15218328/s1, Table S1: Best hyperparameter configuration after nested cross-validation (NCV) process for each machine learning model with the Genetic Algorithm; Table S2: Best hyperparameter configuration after nested cross-validation (NCV) process for each machine learning model with the Bayesian Optimization process.
