# Supplementary material for: Predicting Early Unplanned Readmission to Critical Care Units Using Machine Learning and Metaheuristic Hyperparameter Optimization: Evidence from a Chilean Public Hospital
Source: J Clin Med. 2026 Oct 26;15(21):8328. doi: 10.3390/jcm15218328 (PMC13764531; doi:10.3390/jcm15218328)
Supplement: Supplementary file 1 [file jcm-15-08328-s001.zip › jcm-4574536-supplementary.pdf]

**Supplementary Material: Predicting Early Unplanned Readmission to Critical Care Units Using Machine Learning and Metaheuristic Hyperparameter Optimization: Evidence from a Chilean Public Hospital**

Table S1: Best hyperparameter configuration after nested cross-validation (NCV) process for each machine learning model with the Genetic Algorithm.

| Random Forest | | | | | |
|---|---|---|---|---|---|
| **Parameter** | **NCV 1** | **NCV 2** | **NCV 3** | **NCV 4** | **NCV 5** |
| **Number of estimators** | 139 | 65 | 42 | 26 | 121 |
| **Criterion** | log-loss | entropy | log-loss | entropy | entropy |
| **Max. depth** | 7 | 6 | 5 | 4 | 1 |
| **Min. samples to split** | 2 | 3 | 5 | 2 | 2 |
| **Min. samples per leaf** | 1 | 1 | 5 | 1 | 12 |
| **Max. features** | sqrt | sqrt | sqrt | sqrt | log |
| **Class weights** | balanced | balanced | balanced | balanced | balanced |
| **XGBoost** | | | | | |
| **Loss** | 0.130321 | 0.961168 | 7.51207 | 1.12665 | 0.564032 |
| **Learning rate** | 20 | 59 | 67 | 71 | 43 |
| **Number of estimators** | 0.144821 | 0.0564596 | 0.442342 | 1 | 1 |
| **Subsample** | 2 | 2 | 3 | 2 | 3 |
| **Min. samples to split** | 1 | 4 | 1 | 5 | 4 |
| **Min. samples per leaf** | 3 | 10 | 6 | 7 | 15 |
| **Max. depth** | balanced | balanced | balanced | balanced | balanced |
| **Class weights** | 0.130321 | 0.961168 | 7.51207 | 1.12665 | 0.564032 |
| **Decision Tree** | | | | | |
| **Criterion** | gini | entropy | gini | log loss | gini |
| **Splitter** | random | best | best | best | best |
| **Max. depth** | 1 | 23 | 10 | 10 | 31 |
| **Min. samples to split** | 2 | 4 | 2 | 2 | 8 |
| **Min. samples per leaf** | 1 | 1 | 1 | 1 | 4 |
| **Max. features** | log | log | sqrt | log | log |
| **Class weights** | balanced | balanced | None | balanced | balanced |
| **SVC** | | | | | |
| **C** | 5.54045 | 19.9571 | 10.3221 | 10.6806 | 20.3427 |
| **Kernel** | rbf | sigmoid | sigmoid | sigmoid | sigmoid |
| **Gamma** | 0.379436 | 0.260189 | 0.196305 | 0.188864 | 0.16843 |
| **Logistic Regression** | | | | | |
| **C** | 28.237 | 26.5336 | 25.6408 | 26.8958 | 30.312 |
| **Regularization** | 0.643288 | 0.699 | 0.738235 | 0.972113 | 0.273078 |

Table S2: Best hyperparameter configuration after nested cross-validation (NCV) process for each machine learning model with the Bayesian Optimization process

| **Random Forest** | | | | | |
|---|---|---|---|---|---|
| **Parameter** | **NCV 1** | **NCV 2** | **NCV 3** | **NCV 4** | **NCV 5** |
| **Number of estimators** | 148 | 162 | 24 | 26 | 200 |
| **Criterion** | entropy | log-loss | log-loss | entropy | entropy |
| **Max. depth** | 12 | 8 | 8 | 10 | 9 |
| **Min. samples to split** | 2 | 4 | 7 | 4 | 2 |
| **Min. samples per leaf** | 1 | 3 | 7 | 2 | 14 |
| **Max. features** | sqrt | log | sqrt | sqrt | log |
| **Class weights** | balanced | balanced | balanced | balanced | balanced |
| **XGBoost** | | | | | |
| **Loss** | exponential | log-loss | log-loss | log-loss | log-loss |
| **Learning rate** | 0.17126813 | 1.21076376 | 12.4903545 | 1.25053698 | 0.89321128 |
| **Number of estimators** | 31 | 66 | 87 | 93 | 43 |
| **Subsample** | 0.18598529 | 0.06079657 | 1 | 1.83300459 | 1.36203145 |
| **Min. samples to split** | 3 | 4 | 3 | 2 | 2 |
| **Min. samples per leaf** | 1 | 5 | 2 | 5 | 1 |
| **Max. depth** | 2 | 6 | 8 | 7 | 13 |
| **Class weights** | balanced | balanced | balanced | balanced | balanced |
| **Decision Tree** | | | | | |
| **Criterion** | gini | gini | gini | log loss | entropy |
| **Splitter** | best | best | best | best | best |
| **Max. depth** | 7 | 25 | 5 | 11 | 40 |
| **Min. samples to split** | 3 | 5 | 2 | 2 | 2 |
| **Min. samples per leaf** | 1 | 1 | 1 | 1 | 1 |
| **Max. features** | log | log | log | log | log |
| **Class weights** | balanced | balanced | balanced | balanced | balanced |
| **SVC** | | | | | |
| **C** | 9.27585534 | 28.2433908 | 12.32818 | 17.9520748 | 37.6512632 |
| **Kernel** | sigmoid | sigmoid | sigmoid | sigmoid | sigmoid |
| **Gamma** | 0.69003969 | 0.39996045 | 0.38381379 | 0.23585274 | 0.26017342 |
| **Logistic Regression** | | | | | |
| **C** | 41.0083665 | 34.6028215 | 46.5285465 | 32.2535385 | 49.4715534 |
| **Regularization** | 0.88698565 | 0.89049945 | 0.92151806 | 1 | 0.2937518 |
